# Genome-wide expression patterns of calcium-dependent protein kinases in *Toxoplasma gondii*

**DOI:** 10.1186/s13071-015-0917-z

**Published:** 2015-06-04

**Authors:** Jin-Lei Wang, Si-Yang Huang, Nian-Zhang Zhang, Jia Chen, Xing-Quan Zhu

**Affiliations:** State Key Laboratory of Veterinary Etiological Biology, Key Laboratory of Veterinary Parasitology of Gansu Province, Lanzhou Veterinary Research Institute, Chinese Academy of Agricultural Sciences, Lanzhou, 730046 Gansu Province People’s Republic of China; Jiangsu Co-innovation Center for Prevention and Control of Important Animal Infectious Diseases and Zoonoses, Yangzhou, 225009 Jiangsu Province People’s Republic of China

**Keywords:** *Toxoplasma gondii*, Calcium-dependent protein kinases (CDPKs), Expression patterns

## Abstract

**Background:**

Calcium-dependent protein kinases (CDPKs) are found in plants and some Apicomplexan parasites but not in animals or fungi. CDPKs have been shown to play important roles in various calcium-signaling pathways such as host cell invasion, egress and protein secretion in *Toxoplasma gondii*. The objectives of the present study were to examine the *T. gondii* CDPK genes expression patterns during different development stages and stress responses.

**Methods:**

We carried out a comprehensive expression analysis of CDPK genes based on previously published microarray datasets, and we also used real time quantitative RT-PCR to study ten *T. gondii* CDPK genes expression patterns under acid, alkali, high temperature and low temperature conditions.

**Results:**

Microarrays analysis indicated that some *Tg*CDPK genes exhibited different expression levels in IFN-γ stimuli conditions or at different developmental stages, suggesting that CDPK genes may play different roles in these processes. Expression profiles under low temperature, high temperature, acid and alkaline indicated that most CDPK may be involved in regulating high temperature, acid and alkaline signaling pathways.

**Conclusions:**

We present a genome-wide expression analysis of CDPK genes in *T. gondii* for the first time, and the mRNA levels change with abiotic and biotic stresses, suggesting their functional roles in these processes. These results will provide a solid basis for future functional studies of the CDPK gene family in *T. gondii.*

**Electronic supplementary material:**

The online version of this article (doi:10.1186/s13071-015-0917-z) contains supplementary material, which is available to authorized users.

## Background

The protozoan phylum Apicomplexa comprises thousands of obligate intracellular parasites, many of which cause significant human and animal health problems. *Toxoplasma gondii* infects approximately one third of the global human population and causes severe disease in immunocompromised patients and pregnant women [1] and *plasmodium falciparum*, the causative agents of malaria cause over 1 million deaths per year [2]. In order to perpetuate infection, parasites need to egress from infected cells and then reinvade uninfected cells. In response to these events, parasites have developed various remarkable regulatory mechanisms for proliferation. Among them, intracellular calcium, the second messenger, plays an important role in various signal transduction cascades, including protein secretion, gliding motility, invasion of and egress from host cell, proliferation and differentiation [3].

In *T. gondii*, treatment with a calcium ionophore increases the frequency of calcium transients, resulting in enhanced secretion of micronemal proteins which are required during both invasion and egress. Conversely, chelation of intracellular calcium inhibits microneme secretion, disrupts motility and cell invasion [4]. Transient changes in intracellular calcium concentration are mediated by different calcium sensors or calcium-binding proteins. Comparison of Apicomplexan genomes reveals numerous EF-hand containing proteins including calmodulin-dependent kinases (CaMKs), centrin (CETN)-caltractin-like proteins, CaM-CETN-like proteins, calcium-dependent protein kinases (CDPKs) and CDPK-like proteins [5]. Among them, CDPKs are the most interesting, because they are the most abundant class of calcium sensors in Apicomplexan parasites, at the same time they are also commonly found in plants and some ciliates, but absent from mammalian hosts [6]. The essential functions of Apicomplexan CDPKs and absent from mammalian hosts have made CDPKs as potential drug targets for controlling Apicomplexan-based diseases.

Increasing evidence suggests that CDPKs control important physiological events in the complex life cycles of Apicomplexan parasites. For example, conditional suppression of *Tg*CDPK1 resulted in a weakening of microneme secretion, parasites gliding motility, host cell invasion and egress abilities [7, 8]. CDPK4 in *Plasmodium berghei*, the orthologue of *Tg*CDPK1, regulates cell cycle progression in the male gametocyte [9]. Genetic disruption of *Tg*CDPK3 has demonstrated that it has a regulatory function in parasite physiology in addition to ionophore induced egress [10–12]. In *Plasmodium*, CDPK1 (The orthologue of *Tg*CDPK3) plays a key role in schizont development, microneme secretion, invasion of erythrocyte and regulating mRNAs to assure timely and stage-specific protein expression [13–15]. A recent study demonstrated that *Tg*CDPK7 were crucial for parasites division, growth and proper maintenance of centrosome [16]. Knock-out of *Pb*CDPK3 leads to a pronounced defect in ookinete transmission to the mosquito midgut epithelium and terminates oocysts production [17, 18]. *Pf*CDPK5 plays an essential role in regulating parasite egress from erythrocytes [19], whereas *Pb*CDPK6 is critical for controlling the sporozoites switch from a migratory to an invasion phenotype [20]. Finally, Sharma *et al.* demonstrated that *Pf*CDPK7 bound to PI(4,5)P2 and controlled parasite development in the erythrocyte [21]. Taken together, these studies suggest CDPKs regulate various biologic functions. However, the accurate CDPKs regulatory mechanisms and their targets remain unclear.

In plants, CDPKs constitute a large multigene family. Recently, genome-wide analyses have identified 34 CDPK isoforms in *Arabidopsis* genome [22], 31 genes in rice genome [23] and 25 CDPK genes in *canola (Brassica napus L.)* [24]. Apicomplexan parasites also contain multiple CDPK genes [25]. Sibley *et al.* indicated *T. gondii* contained 12 CDPKs gene [5], While Tallevich *et al.* identified TGME49_240390 on the Toxodb database as a novel CDPK [26]. Through our unpublished data, this CDPK contains 3 EF-hand, and don’t have known orthologues in Apicomplexan parasites (except for *Neospora caninum*). Here, we named it as *TgCDPK10*. To our knowledge, only three *Tg*CDPKs (CDPK1, CDPK3 and CDPK7) have been studied, yet the physiological functions of the majority of CDPKs remained unclear.

In the present study, we carried out a comprehensive expression analysis of calcium-dependent protein kinase genes based on previously published microarrays datasets. Furthermore, we also studied ten *T. gondii* CDPK genes expression patterns under acid, alkali, high temperature and low temperature conditions. Our results showed most *TgCDPKs* may regulate stress responses.

## Methods

### Parasite cultures

*T. gondii* RH strain were maintained by passage through Vero cell monolayers in Dulbecco’s modified Eagle’s medium (DMEM) with 2 % fetal calf serum (FCS), 10 mM HEPES (pH = 7.2), 100 U/ml penicillin and 100 Ug/ml streptomycin at 37 °C with 5 % CO2 as previously described [27].

### Stress treatments

Freshly released tachyzoites were harvested by centrifugation (1000 *g* × 4 min). Parasites were washed with phosphate-buffered saline (PBS) to remove impurities, and then replaced with fresh control or medium with different pH value. A low temperature treatment was carried out at 4 °C under the same conditions while the high temperature treatment was at 42 °C. Acid and alkaline solution treatment were carried out by cultivation in DMEM with acetate buffer (pH3.6) or 50 mM Hepes (pH 8.1) at 37 °C in the presence of air.

### Real-time quantitative RT-PCR

Total RNA was isolated from parasites of different time periods using Trizol reagent according to the manufacturer’s instructions (Invitrogen, Carlsbad, CA, USA). The properties of RNA samples were examined by Goldview-stained agarose gel electrophoresis and spectrophotometric analysis. Total RNA samples were transcribed into cDNA using superscript First-Strand Synthesis System (Promega, USA). Real-time quantification RT-PCR was performed in 20 ml volume reaction mixture containing 2 × SYBR Green qPCR Supermix (Invitrogen, Carlsbad, CA, USA), 10 mM gene-specific primer and 0.1 mg of cDNA. The thermal cycling conditions were set as follows: 95 °C for 3 min followed by 40 cycles of amplification at 95 °C for 15 s, 60 °C for 31 s and 72 °C for 15 s. Lack of primer dimers or genomic DNA contamination of reagents was verified with melting curve analysis. The *β*-tubulin gene was used as internal reference for all the reactions analysis and the primers used for qRT-PCR are described in Additional file 1: Table S1 [28]. The relative gene expression levels were determined using 2^-△△^CT method and each experiment was performed three biological replicates.

### Microarray analysis

To examine the global expression profiles of CDPK genes among different invasion periods, cell cycle, developmental stages and on IFN-γ-dependent cell mediated immune response, expression analysis were generated using published microarrays data which were deposited at NCBI Gene Expression Omnibus (GEO) database under the series accession GSE20480, GSE19092, GSE32427, and ArrayExpress database E-MEXP-3579 respectively. Dendrogram and heatmap for display expression pattern were carried out using Cluster 3.0 for normalizing and hierarchical clustering with average linkage. The analyzing datasets was visualized by Java Tree-View 1.1 program.

## Results and discussion

### Expression analysis of CDPK genes by microarray

The published microarray data provided us abundant resources for studying *Tg*CDPKs gene expression patterns. We obtained microarrays data from four microarrays experiments [29–33]. Fortunately, all of the *T. gondii* CDPK genes have the corresponding probe sets in these datasets.

**Fig. 1:**
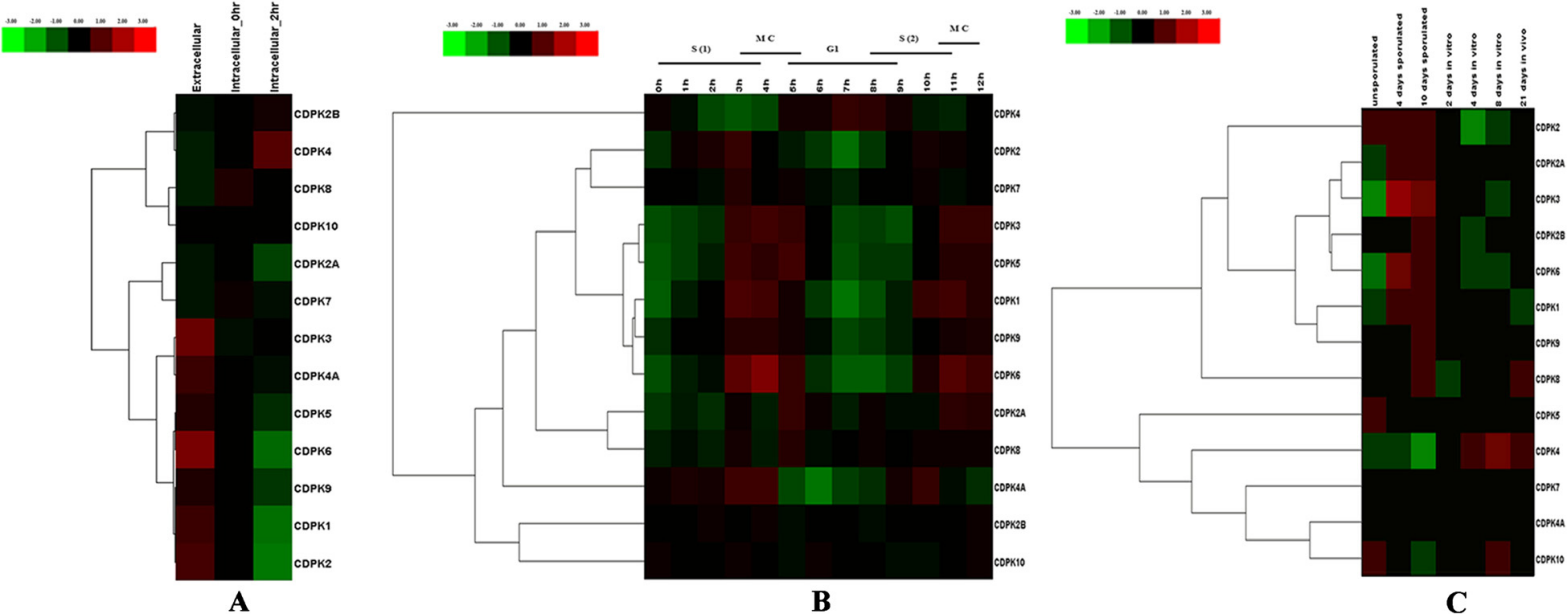
**a**. Expression profile clustering of *Toxoplasma gondii* CDPK genes during host cell invasion. Extracellular: Harvested from outside the HFF cell. Intracellular_0hr: Harvested from inside the HFF cell, shortly after invasion; Intracellular_2hr: Harvested from inside the HFF cell, 2 h after invasion. Two biological replicates were performed each experiment. The colored bar representing the relative signal intensity values is shown at the top of the chart. Hierarchical clustering was used in data analysis. **b**. Expression profile clustering of *Toxoplasma gondii* CDPK genes across cell cycle expression profiles in HFF cell. h: the time post-thymidine release. Two biological replicates were performed each experiment. The colored bar representing the relative signal intensity values is shown at the top of the chart. Hierarchical clustering was used in data analysis. **c**. Expression profile clustering of *Toxoplasma gondii* CDPK genes across oocyst, tachyzoite and bradyzoite developmental stages. Oocysts were harvested from kitten feces and purified for RNA extraction at Day 0 (unsporulated), Day 4 post-induction of sporulation (mid-sporulation) and Day 10 post-induction of sporulation. *In vitro*, 2 dpi tachyzoite, 4 and 8 dpi bradyzoite samples are from separately infected cultures of HFF cell. *In vivo*, 21 dpi bradyzoite cysts harvested from the brains of mice that were infected with oocysts. 2 biological replicates each. The colored bar representing the relative signal intensity values is shown at the top of the chart. Hierarchical clustering was used in data analysis

To investigate the expression patterns of CDPK genes in *T. gondii* when it transforms from an extracellular to an intracellular environment, a microarray of three periods of parasites (extracellular parasites, newly invasion parasites and intracellular parasites) were performed [29]. A heatmap representation of the expression profiles for 13 CDPK genes during parasite invasion is shown in Fig. 1a. CDPK1, CDPK2, CDPK3, CDPK4A, CDPK5, CDPK6 CDPK9 were down-regulated in intracellular 2h parasites. For instance, the expression patterns of CDPK6, CDPK2 and CDPK1 have predominantly decreased 6.4-fold, 4.7-fold and 4.0-fold, respectively. It has been demonstrated that CDPK1 was involved in host cell invasion, parasites motility and egress [7], the expression patterns of CDPK6 and CDPK2 are similar to that of CDPK1 suggesting that CDPK6 and CDPK2 may be involved in host cell invasion. The expression of CDPK2A, CDPK2B, CDPK7, CDPK8 and CDPK10 were quite stable, the differences of expression were less than 1.5-fold, suggesting that these genes are not responsible for invasion but probably possess other functions, for example CDPK7 is involved in parasite division [16]. As shown in Fig. 1a, the transcript level of CDPK4 increased 2.4-fold in intracellular 2h stage comparing to intracellular 0h stage, which may reflect that CDPK4 may have some novel functions rather than just being involved in invasion.

To investigate the expression profiles of *Tg*CDPK genes in the intracellular cell cycle, another microarray data set [30] was used for this analysis. In this experiment, *T. gondii* were synchronized by thymidine block, and then cell cycle expression profiles were studied after thymidine release. As shown in Fig. 1b, the expression levels of CDPK1, CDPK2, CDPK3, CDPK4A, CDPK5, CDPK6 and CDPK9 were rather higher from S phase to cytokinetic periods (S/M phases), and then became lower in the major G1 period, especially for CDPK6 and CDPK1 which decreased 5.9-fold and 4.4-fold, respectively. CDPK4 had a different expression pattern with a lower expression in S/M phases but a higher expression in G1 period. The expression levels of CDPK2A, CDPK2B, CDPK7, CDPK8 and CDPK10 genes were quite stable, and there was no obvious change during the intracellular cell cycle. Comparison of the results of two microarrays revealed an alignment between CDPK genes elevated in extracellular parasites and the S/M phases and down-regulated in the intracellular mRNA expression with the G1 period. The expression level changes of CDPK1, CDPK2, CDPK3, CDPK4A, CDPK5, CDPK6 and CDPK9 were correlated with known microneme proteins which are involved in host cell invasion, suggesting these CDPKs may also be involved in host cell invasion. In extracellular parasites, the expression of proteins involved in invasion, motility and signal transduction are increased and the parasites appear to be optimally primed for cell invasion. Conversely, the expressions of biosynthetic and metabolic genes are increased after *T. gondii* entry and in the G1 period suggesting parasites begin to proliferate [29]. Thus, these results indicated that CDPK1, CDPK2, CDPK3, CDPK4A, CDPK5, CDPK6, and CDPK9 may be involved in host cell invasion, egress and motility, while CDPK4 may play novel roles in metabolism and DNA replication.

To study the function of *Tg*CDPKs in specific parasite development stages, a third microarray data [31, 32] was used to carry out this study. As show in Fig. 1c, the expression levels of CDPK3 and CDPK6 were lower in unsporulated oocysts but higher in sporulated oocysts of 4 days, and the expression became lower as the development of oocysts increased. The expression levels of CDPK3 and CDPK6 were 6.6-fold and 2.8-fold in d4 oocysts comparing to d0 oocysts, respectively; the expression levels of CDPK3 and CDPK6 were 3.9-fold and 1.7-fold in d10 oocysts comparing to d0 oocysts, respectively. These results suggested that CDPK3 and CDPK6 may be involved in oocysts development or adjust oocysts to suit the nutrient-poor and stressing external environment. The expression levels of other CDPKs were almost unchanged during this process, and the expression changes of these CDPK genes may need some other stimuli.

### Expression profiles of the *T. gondii* CDPK genes under different stresses

Most bacterial pathogens significantly alter their transcriptomes to fine-tune the infected host cell environment and thus avoid clearance by host-cell-intrinsic mechanisms and achieve efficient invasion to the next host. *T. gondii* has also evolved various strategies to modify the host environment, including altering the gene expression and reshaping the host genome expression to responses to intracellular environmental changes [34, 35].

To identify the effects of CDPKs on IFN-γ-dependent cell mediated immune response, the expression of the *Tg*CDPK genes were analyzed in C57BL/6 mice, IFN-γ KO mice (Parasites were harvested from the peritoneal cavity of the mice after 4 days with 2 × 10^6^ parasites intraperitoneally) and *in vitro* environment in HFF cells (Parasites were harvested 48 h later as parasites were beginning to egress) using the published microarray data with four biological replicates [33]. The results indicated that the expression levels of all CDPK genes were quite similar in IFN-γ KO mice and *in vitro* samples, which the expression changes were less than 2-fold. The results suggest that the functions of *Tg*CDPKs in IFN-γ KO mice and in HFF cells are not significantly different. CDPK2B, CDPK7 and CDPK10 were significantly up-regulated and CDPK6 was down-regulated *in vitro* comparing to that in WT mice. The up-regulated CDPK genes CDPK2B, CDPK7 and CDPK10 increased 2.8, 4.6 and 2.4-fold, respectively and the down-regulated CDPK6 gene declined 2.5-fold. Similar results were obtained from comparison between *in vivo* in WT and in IFN-γ KO mice. This indicates that the changes of four CDPK genes expression patterns are largely induced in response to the IFN-γ-dependent-innate immune response, which suppresses *T. gondii* proliferation. It has been reported that knock-down of *Tg*CDPK7 protein resulted in pronounced defects in parasite division [16]. These suggest that CDPK7 gene may increase expression in response to avoid IFN-γ-induced suppression of *T. gondii* proliferation. Thus, these results suggest that the four CDPK genes may play an important role in IFN-γ-dependent-innate immune response. Further studies are needed to explain the accurate processions.

**Fig. 2:**
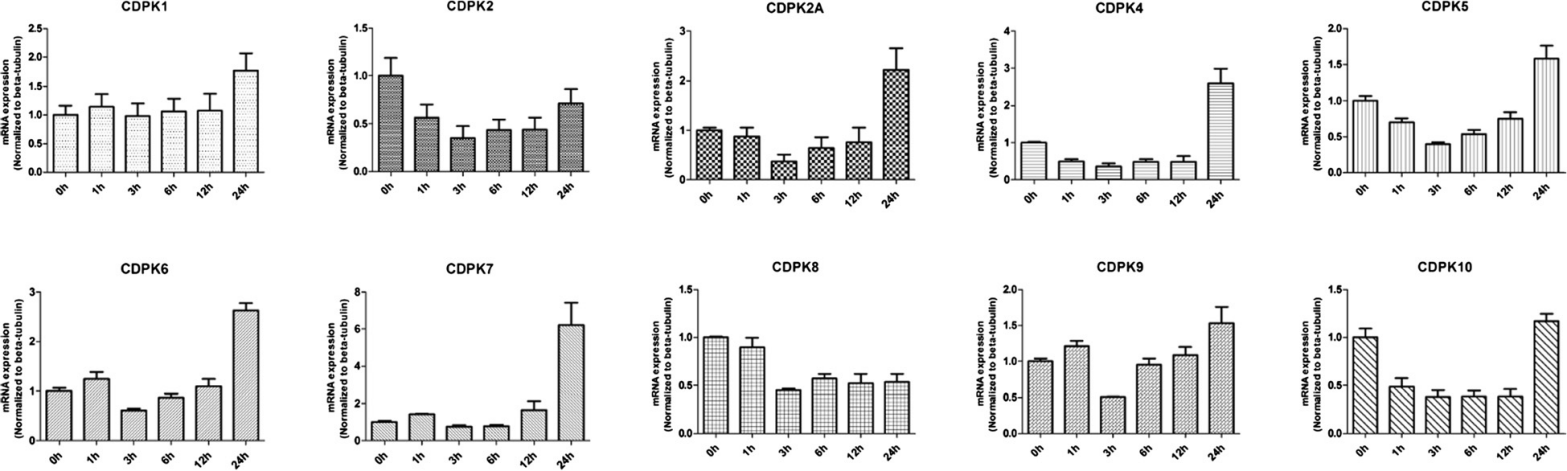
Expression profile clustering of *Toxoplasma gondii* CDPK genes exposed to acid DMEM (pH 3.6) for various times as indicated by quantitative real-time RT-PCR analysis. Each bar represents the mean ± SD values (*n* = 3)

Multiple studies had demonstrated that many plant CDPKs regulate biotic and abiotic stress responses, including temperature, salt and drought stresses [36, 37]. When tachyzoites enter host, they may meet different abiotic stress such as acid in stomach, alkaline in intestine and high temperature when the host runs a fever. To examine the effects of CDPK genes expression patterns under these stress responses, freshly released tachyzoites were treated under conditions of low temperature (4 °C), high temperature (42 °C), acid DMEM (pH 3.6) and alkaline DMEM (pH 8.1), respectively. We did Real-time quantitative RT-PCR to detect the expression level of *T. gondii* CDPKs. As shown in Fig. 2, acid solution (pH 3.6) treatment caused a slight alteration in the expression levels of CDPK1 and the largest gap was less than 2-fold, however, the transcript levels of other CDPKs had a marked decrease at 3 h and then soon increased. For example, the transcript levels of CDPK2, CDPK5, CDPK8 CDPK9 and CDPK10 were almost equal to untreated levels at 24 h, for example CDPK2 was down-regulated 2.8-fold at 3 h and then slightly recovered to untreated levels. The transcript abundance of CDPK2A and CDPK4 increased higher than untreated levels at 24 h. The expression level of CDPK2A was 2.7-fold lower at 3 h and 2.2-fold higher compared to untreated, respectively. Finally, the transcript levels of CDPK6 and CDPK7 remain unchanged during 3 to 12 h, while increased 2.6 and 6.2-fold at 24 h, respectively. Although there was no study to show the specific functions related to the changes of the expression pattern, the over-expression of CDPKs may be involved in acid resistance.

**Fig. 3:**
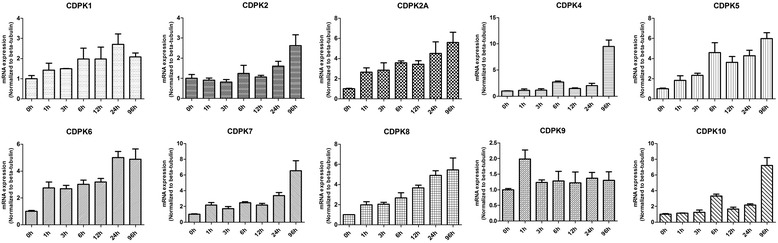
Expression profile clustering of *Toxoplasma gondii* CDPK genes exposed to alkaline DMEM (pH 8.1) for various times as indicated by quantitative real-time RT-PCR analysis. Each bar represents the mean ± SD values (*n* = 3)

**Fig. 4:**
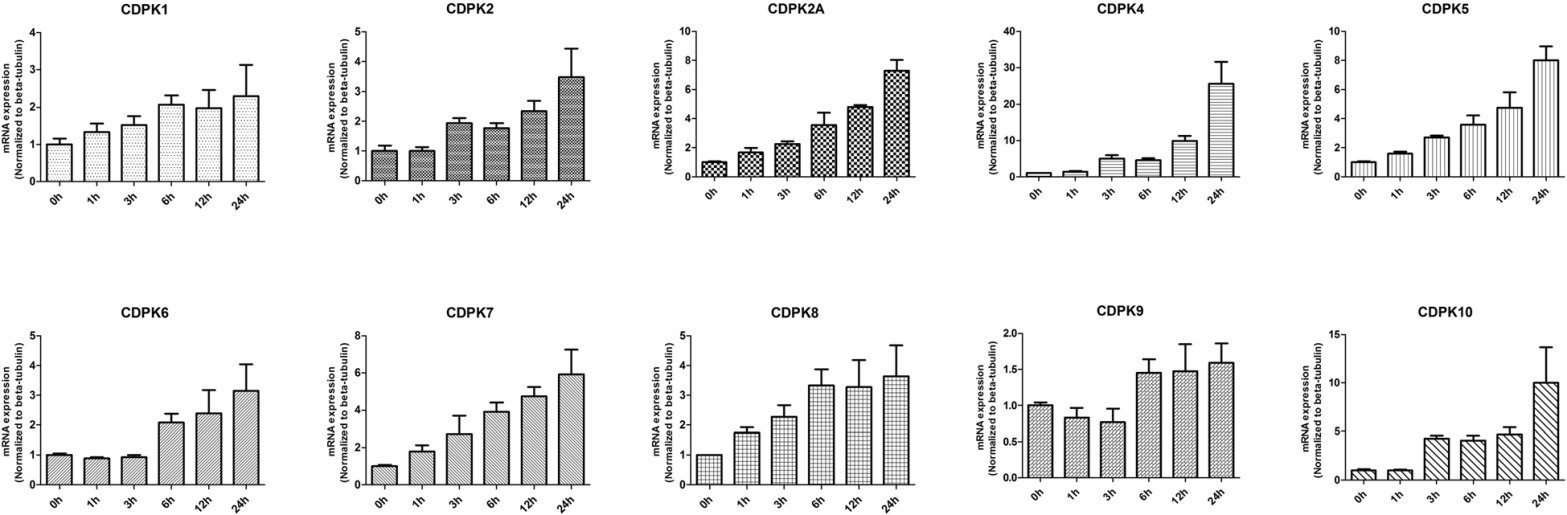
Expression profile clustering of *Toxoplasma gondii* CDPK genes exposed to high temperature (42 °C) for various times as indicated by quantitative real-time RT-PCR analysis. Each bar represents the mean ± SD values (*n* = 3)

Under alkaline treatment (Fig. 3), the expression patterns of CDPK9 remain unchanged, suggesting that CDPK9 may have nothing to do with alkaline responses. However, alkaline treatment caused a marked increase in the transcript abundance of 9 other CDPK genes, but the patterns of transcription changes were different among the CDPKs. For example, CDPK2A, CDPK6 and CDPK7 were increased between 2.2 and 2.7-fold at 1 h; CDPK2, CDPK4, and CDPK10 were remained unchanged at 1 h, but were significantly up-regulated between 5.6 and 9.5-fold at 96 h, and the expression of CDPK1, CDPK5 and CDPK8 were increased steadily. Prior work has suggested that bradyzoite differentiation relate to cell cycle, with the first detectable initiation of the differentiation program occurring in S/M phase, from our results we can draw the same conclusion, the CDPK genes (except for CDPK9) may relate to the bradyzoite differentiation. Our results indicated CDPK genes expression may help *T. gondii* adapt to alkaline environment such as in the intestine. Some plants CDPK play an important role in temperature stresses [36, 37]. In order to examine the functions of *T. gondii* CDPKs in response to temperature stresses, we treated *T. gondii* in different temperatures and then analyzed the CDPKs expression patterns. As show in Fig. 4, all the CDPKs (except CDPK9) were up-regulated in response to high temperature stress. For example, CDPK1, CDPK2, CDPK2A, CDPK5, CDPK6, CDPK7, CDPK8, CDPK10 and CDPK4 were found up-regulated between 2.3 and 25.5-fold, at 24 h after high temperature treatment. Expression of CDPK4 and CDPK10 had a marked increase at 3 h, whereas other genes increased steadily. However, the CDPK9 transcription level was kept quite stable and the expression change was less than 2-fold during 24 h. When the host is infected with *T. gondii*, fever is one of the important reactions to the infection. *T. gondii* may change CDPKs expression to resist the high temperature of the hosts.

In the present study, when *T. gondii* were treated under low temperature conditions, the results indicated that the expression levels of all *TgCDPKs* remain unchanged (As shown in Additional file 2: Figure S1). Many plant CDPKs have been reported to play crucial roles during cold stress [36]. In contrast our results suggested that *TgCDPK*s does not play such a role. This may be due to the fact that *T. gondii* do not encounter a cold environment in the host.

## Conclusions

We conducted a comprehensive expression analysis of the CDPK gene family in *T. gondii* for the first time. We found the following results: First, during the *T. gondii* invasion and in the intracellular cell cycle, the expression pattern of CDPK4 was distinctly different from others. Second, in parasite development stages, only CDPK3 and CDPK6 change their expression level during oocyst sporulation. Third, CDPK2B, CDPK7 and CDPK10 were significantly up-regulated and CDPK6 was down-regulated during the IFN-γ stimulate. Fourth, during the acid solution treatment, all CDPKs had a marked decrease at 3 h and then increased except CDPK1 which remain unchanged, and during the alkaline treatment, CDPK9 remain unchanged while the other CDPK genes had a marked increase in the transcript levels. Finally, during temperature stresses, all the CDPK (except CDPK9, which remain unchanged) were up-regulated in response to high temperature stress, while the expression patterns of all the CDPK genes remain unchanged in response to cold stress. Our results suggest CDPKs are a large family of multi-functional genes which may play essential roles in parasite development, abiotic and biotic stress responses. These results will provide a solid basis for future functional studies of the CDPK gene family in *T. gondii.*

## Additional files

Additional file 1: Table S1. PCR primers used in this study. Additional file 2: Figure S1. Expression profile clustering of *Toxoplasma gondii* CDPK genes exposed to cold temperature (4 °C) for various times as indicated by quantitative real-time RT-PCR analysis. Each bar represents the mean ± SD values (*n* = 3).
