# Supplementary material for: Genome-wide expression patterns of calcium-dependent protein kinases in Toxoplasma gondii
Source: Parasit Vectors. 2015 Jun 4;8:304. doi: 10.1186/s13071-015-0917-z (PMC4459671; doi:10.1186/s13071-015-0917-z)
Supplement: Additional file 1: Table S1. — PCR primers used in this study. [file 13071_2015_917_MOESM1_ESM.doc]

**Table S1** PCR primers used in this study

| Gene | | Forward primer | Reverse primer |  |
| --- | --- | --- | --- | --- |
| DPK1 | | CTACACGAAGGAGCAGATCA | AGTTCCTTCGTTTCGTCTTG |  |
| CDPK2 | | GGTTCACCAGGGTGTCTTCA | CGAAGTCGATGAGCTTGATG |  |
| CDPK2A | | TGCGCAGCATATGTCAGA | GCGAACTTCTTGAACTGACAAC |  |
| CDPK4 | | AGACAAGAGCGGAGGAGATCA | CGTTCAAAGGCCTCTCGAAT |  |
| CDPK5 | | AGGCATCCGTGGATTCAGT | CAGCTTGTGCAGTCTCTGAAA |  |
| CDPK6 | | AAGGCTACGTTGCTGAGTTG | AGTTCTGCGAGACCGAAGTC |  |
| CDPK7 | | GGCGTGATTATGTACCTCCTT | GAAGCATGCGGACAATGAG |  |
| CDPK8 | | AGACGGAAGCGGAAACATC | CACCAAGCCGTCAAAACTAC |  |
| CDPK9 | | GACAACGAGGAAACGGTAAA | GGACCAAGTCTTTTGCTTGA |  |
| CDPK10 | | AAGGTGGATCTCGAGCGAAA | GCCCTTGTTGTGGAGTGTGT |  |
| *β*-tubulin | | GTCTCCACTTCTTCCTCATTG | GTTCTTTGCGTCGAACATC |  |
|  |  | | | |
