# Supplementary figures and images for: Genome-wide expression patterns of calcium-dependent protein kinases in Toxoplasma gondii
Source: Parasit Vectors. 2015 Jun 4;8:304. doi: 10.1186/s13071-015-0917-z (PMC4459671; doi:10.1186/s13071-015-0917-z)

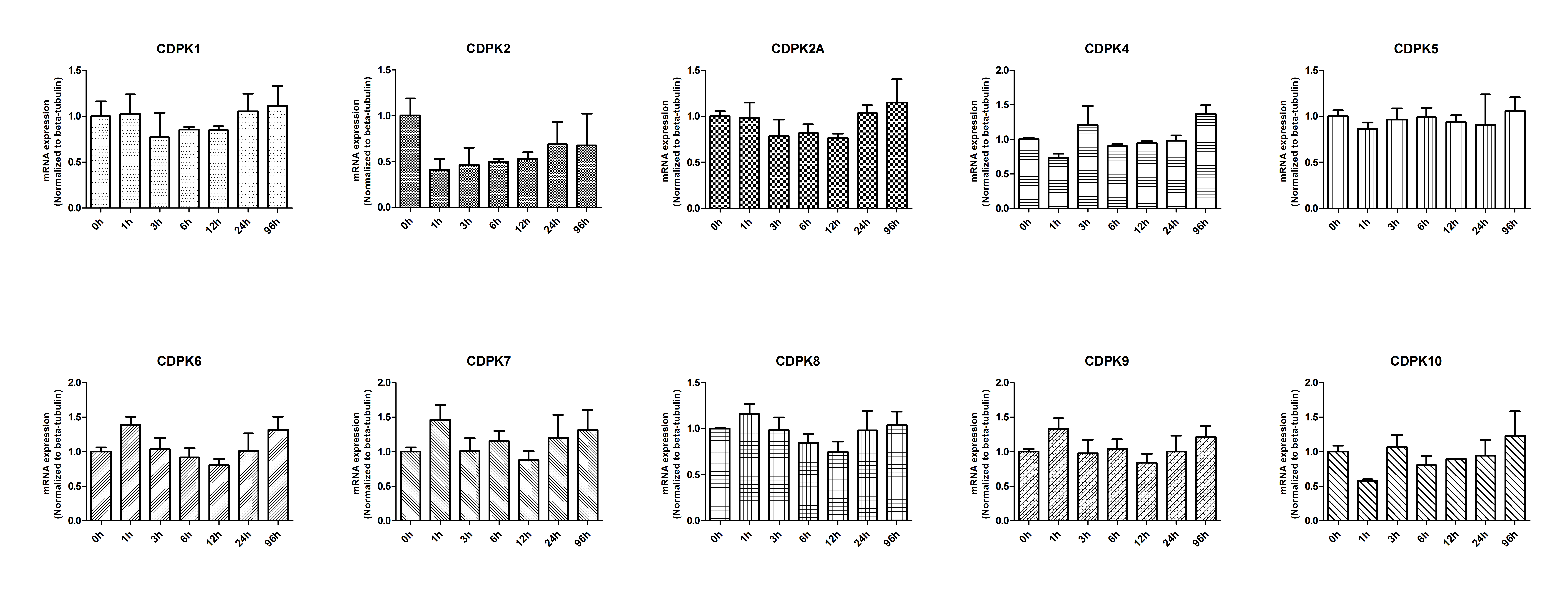

Supplement: Additional file 2: Figure S1. — Expression profile clustering of Toxoplasma gondii CDPK genes exposed to cold temperature (4 °C) for various times as indicated by quantitative real-time RT-PCR analysis. Each bar represents the mean ± SD values (n = 3). [file 13071_2015_917_MOESM2_ESM.tif]
